# Evolving Strategies, Opportunistic Implementation: HIV Risk Reduction in Tanzania in the Context of an Incentive-Based HIV Prevention Intervention

**DOI:** 10.1371/journal.pone.0044058

**Published:** 2012-08-27

**Authors:** Laura Packel, Ann Keller, William H. Dow, Damien de Walque, Rose Nathan, Sally Mtenga

**Affiliations:** 1 Global Health Sciences, University of California San Francisco, San Francisco, CA, USA; 2 School of Public Health, University of California, Berkeley, Berkeley, CA, USA; 3 World Bank Development Research Group, The World Bank, Washington DC, USA; 4 Ifakara Health Institute, Dar es Salaam, Tanzania; Rollins School of Public Health, Emory University, United States of America

## Abstract

**Background:**

Behavior change communication (BCC) interventions, while still a necessary component of HIV prevention, have not on their own been shown to be sufficient to stem the tide of the epidemic. The shortcomings of BCC interventions are partly due to barriers arising from structural or economic constraints. Arguments are being made for combination prevention packages that include behavior change, biomedical, and structural interventions to address the complex set of risk factors that may lead to HIV infection.

**Methods:**

In 2009/2010 we conducted 216 in-depth interviews with a subset of study participants enrolled in the RESPECT study - an HIV prevention trial in Tanzania that used cash awards to incentivize safer sexual behaviors. We analyzed community diaries to understand how the study was perceived in the community. We drew on these data to enhance our understanding of how the intervention influenced strategies for risk reduction.

**Results:**

We found that certain situations provide increased leverage for sexual negotiation, and these situations facilitated opportunistic implementation of risk reduction strategies. Opportunities enabled by the RESPECT intervention included leveraging conditional cash awards, but participants also emphasized the importance of exploiting new health status knowledge from regular STI testing. Risk reduction strategies included condom use within partnerships and/or with other partners, and an unexpected emphasis on temporary abstinence.

**Conclusions:**

Our results highlight the importance of increasing opportunities for implementing risk reduction strategies. We found that an incentive-based intervention could be effective in part by creating such opportunities, particularly among groups such as women with limited sexual agency. The results provide new evidence that expanding regular testing of STIs is another important mechanism for providing opportunities for negotiating behavior change, beyond the direct benefits of testing. Exploiting the latent demand for STI testing should receive renewed attention as part of innovative new combination interventions for HIV prevention.

## Introduction

Traditional behavior change communication interventions, while still a necessary component of HIV prevention, have not in and of themselves been shown to be sufficient to stem the tide of the epidemic [1]. In light of recent research findings showing the effectiveness of male circumcision and early initiation of anti-retroviral therapy (ART) in preventing HIV, the prevention landscape has shifted away from behavioral interventions toward biomedical interventions, and has placed a central importance on HIV testing as a gateway to more evidence-based HIV prevention interventions [2] .

The shortcomings of behavioral change interventions are at least in part due to barriers arising as a result of structural or economic constraints on behavior change [3], [4]. Arguments are now being made for combination prevention packages that include behavior change, biomedical, and structural interventions in an effort to address these barriers along with the complex, multilayered set of vulnerabilities and risk factors that may lead to HIV infection [1], [2], [5], [6]. Additionally, incentive-based interventions that stimulate demand for HIV testing and knowledge of HIV status among both individuals and couples, potentially increasing referrals into male circumcision and/or treatment, are increasingly being explored [7], [8]. Interventions that address the structural barriers to behavior change and have the potential to increase the effectiveness of behavioral change interventions are also on the rise [8], [9].

Qualitative data from one such structural intervention in Tanzania, the RESPECT trial [9], provides a unique opportunity to learn more about sexual behavior change strategies and implementation in the context of a monetary incentive to engage in safer sex strategies. Our hypothesis is that an economic incentive to avoid unsafe sex, along with regular testing for sexually transmitted infections (STIs), may work in combination with traditional behavior change messages to overcome some of the barriers inherent in sexual behavior change, especially for women. In particular, we explore how different components of the RESPECT intervention may have created both new leverage in sexual negotiations, as well as increased opportunities for exercising that leverage.

We know from previous research that individuals are frequently employing risk reduction strategies, but often not in the ways that dovetail with the traditional sexual behavior change messages [10]–[13]. For example, strategies of married women often center around convincing their husbands to leave outside partners or to use condoms with outside partners, while male strategies are focused on careful partner selection and condom use with non-primary partners [11].

Risk reduction strategies such as condom use and reducing the number of sexual partners may be implemented inconsistently as a result of structural and cultural factors including gender inequality [12], [14]–[16], women's economic dependence on men, norms of marriage [17]–[20], [21], desire to have children [22]–[25], difficulty in assessing risk of partners [10]–[13] and fear of gender-based violence [26], [27]. In fact, studies done in Tanzania and Kenya revealed that married women sometimes fail to disclose their HIV status to their partners due to fear of abandonment, accusations, physical violence and loss of confidentiality, potentially leading to HIV transmission within marriage [28], [29]. In two studies from Tanzania, only 17% and 40% of the women testing positive for HIV, respectively, had disclosed their HIV status to their partner, even after a considerable follow-up period [30], [31]. The main reasons for not disclosing HIV status were fear of stigma and divorce, fear of losing confidentiality, women's lack of decision-making power, communication patterns between partners and male partners' attitudes to HIV voluntary counseling and testing [30], [31].

Less research has been conducted on behavior change barriers that might exist for men, in large part because the epidemiology shows higher HIV prevalence among women. Notably, one recent exception from the comparative anthropology perspective discusses in detail the “opportunity structures” in place that perpetuate the benefits that men derive from having multiple and extramarital partners [32]. While barriers to behavior change for men specifically is not discussed here, it is important to note that for men, as for women, benefits derived from engaging in risky behaviors may outweigh the perceived risks of doing so.

Using qualitative data from a trial of economic incentives for testing STI negative, in this paper we explore how a cash incentive, in combination with regular STI testing, influenced strategies for risk reduction. This is important to understanding behavioral mechanisms through which the RESPECT trial led to reduced STIs. The cash awards are a type of structural intervention, which we hypothesized could lead to behavioral change by both men and women, but it was a priori unclear what type of behavioral change strategies would be adopted and how. In addition, the study's provision of regular STI testing (in an environment in which STI testing was generally not available on demand) allowed analysis of the mechanisms through which STI testing could operate. STI testing is understood to reduce risks through epidemiological pathways by identifying treatment needs, but in this paper we explore whether the expanded health status knowledge from STI testing led RESPECT participants to change sexual behaviors, and if so, how. Our investigation also helps to understand how the structural cash intervention and the testing component could work synergistically as part of a combination prevention package that can assist men and women with opportunities to better act on behavioral change intentions, thus potentially increasing the effectiveness of traditional behavior change interventions.

## Methods

The data reported on in this paper come from the qualitative component of the RESPECT study. Detailed methods of the RESPECT study have been reported elsewhere [9], but briefly, the study was a three-arm randomized trial testing the effectiveness of a cash incentive conditional on testing negative for a panel of curable STIs. The intervention arm was divided into two sub-arms – a low-value award arm eligible for up to $30 over the course of the study (approximately $10 per testing round), and a high-value award arm eligible for up to $60 (approximately $20 per testing round). Those amounts were determined based on focus-group discussions in neighboring villages conducted before the intervention started, balancing sufficient incentive levels against concerns about scalability and potential coercion. All participants were tested for STIs at baseline and then every 4 months for one year, and provided free treatment if they tested positive. Participants in the two intervention arms were eligible to receive award incentive payments if they tested negative for curable STIs at the 4, 8, and 12-month testing rounds. Inclusion criteria consisted of males and females, aged 18–30 (and spouses ages 16 or over), residing in one of 10 study villages within the Kilombero/Ulanga districts of the Ifakara Health and Demographic Surveillance System (IHDSS) in south-west Tanzania [33].

The use of cash incentives to encourage sexual behavioral change is an innovative approach, but not without controversy. The background and justification of the RESPECT design are discussed in more detail [34]. For a more general discussion on the ethics of incentives for health promotion, particularly in low-income settings, see London et al [35]. The one-year RESPECT intervention resulted in a 27% reduction in STIs in the high-value cash award arm as compared to the control arm [9] thus appeared to impact sexual behavior. The accompanying qualitative interviews were designed to elucidate the constraints on sexual behavior change and the strategies used by participants to effectuate change.

The qualitative study participants were recruited from four of the ten villages that were participating in the RESPECT study. The four qualitative villages represented a range of semi-urban to more rural, and ranged from 15 minutes to a 2-hour drive to Ifakara, the main urban center in the district. We used stratified random sampling to select the qualitative study participants at baseline. In each village, the strata of interest were gender, marital status, and intervention/control group. We over-sampled from the treatment group as we were interested in hearing more experiences relating to how the money did or did not motivate sexual behavior change. This analysis utilizes data from in-depth interviews conducted at the baseline, 4-month and 8-month study visits. At baseline, we randomly sampled 92 trial enrollees (43 men, 49 women) from four of the ten study villages to be interviewed. Of these 92, 80 showed up at the study site to pick up their STI results at the next study visit three weeks later, at which time the qualitative interviews were conducted. Those who did not show up to the study site were more likely to be male (p = 0.08), but there were no other significant differences. Of these 80 that were interviewed, 66 transcripts were received (14 transcripts were lost through data management error–either the recordings were inadvertently deleted or the electronic version of the transcript was inadvertently deleted).

At the 4 and 8-month study visits new qualitative participants were purposively sampled in order to increase the number of participants interviewed who had tested positive for an STI. In addition, we intentionally conducted fewer overall interviews at 8 months because we had reached saturation with the number of participants we had already enrolled. As a result, some participants who were interviewed in 4 months were purposely not interviewed in 8 months. The decision of which participants to drop was not random; transcripts were reviewed to screen out the least cooperative respondents who were not deemed to be particularly candid, and consideration was given also to gender, intervention group, and STI status of the participant. Transcripts were not reviewed for content when making the decision to keep or drop a respondent; rather the text was reviewed for coherency of responses and a willingness to talk at relative length about the subject matter. No participants who were recruited for the qualitative study from those coming to the study station to pick up test results refused to be interviewed, however, some targeted qualitative respondents did not return to the study station for the follow up visits at months 4 (4.6%) and 8 (1.8%).

Qualitative participants received a small cash payment equal to approximately $3 USD at the end of each interview to reimburse them for transport and extra time spent at the study station. Interviews took place at the study station in tents or secluded areas.

All interviews were audio-recorded, transcribed in Kiswahili, and then translated into English. The interviews were conducted using an interview guide. The main topic areas covered during the interviews were opinions about the study, community perceptions about the study, strategies and/or steps for avoiding STI and getting the cash award, perceptions of the cash incentive, and future plans generally and for use of the cash incentive if received. The guide was revised for each follow-up visit (months 4, 8 and 12) and included questions about respondent's experiences being enrolled in the study over the previous four months, what strategies they tried, and why or why not these strategies were successful.

Following a methodology developed by Watkins and Swidler termed conversational journals, we also hired ten diarists who were “cultural insiders” in all of the communities in which the RESPECT trial took place [36]. Swidler and Watkins have very successfully used this method as part of their HIV research in Malawi [36]. Diarists were paid $30 per filled notebook (limit one notebook per month). Data collected from the conversational journals does not include names or identifying information. The data analyzed for this manuscript includes diaries from February through July 2009, from all ten study villages.

Qualitative analysis was conducted using a phenomenological approach – relying on in-depth descriptions by study participants to derive meaning and understanding of experience [37]. English transcripts of interviews and diary entries were imported into TAMS Analyzer Qualitative Coding Software. We developed codes iteratively for strategies used by study participants or discussed by diarists to avoid unsafe sex or to protect themselves from HIV or STI infection based on descriptions from study participants. Codes were then grouped into clusters by similarity of strategy, and all transcripts were then coded with TAMS Analyzer using this set of strategy codes. We then used the software to compare and analyze strategies discussed by intervention group and by gender. The demographic and other quantitative data included here came from the structured questionnaire conducted at the baseline study visit, and administered to all study participants. Differences in demographic and other characteristics noted here were examined using a Chi Square test, and differences were considered significant if p<0.05.

The study was approved by the Institutional Review Boards of the Ifakara Health Institute, the University of California at Berkeley, and by the Tanzania National Institute for Medical Research. All participants provided written informed consent.

## Results

*Qualitative Study Population*, for a total across the whole study of 216 interviews, representing 111 individuals. Table 1 shows the same demographics and other characteristics for the qualitative study population at baseline, 4 months, and 8 months, in addition to the overall study population at baseline. Notable differences in the qualitative and quantitative populations included the distribution by intervention group (oversampling of those in the treatment groups was intentional), and marital status (the proportion of married participants was lower in the qualitative population).

**Table 1:** Characteristics of total study population at baseline and qualitative study population.

Variable	All Study Participants at Baseline (%)	Qualitative Participants at Baseline (%)	Qualitative Participants at 4-month (%)	Qualitative Participants at 8-month (%)
	(N = 2399)	(N = 66)	(N = 95)^a^	(N = 55)^b^
Female	50.3	57.6	61.1	56.4
Age (mean)	26.4 (se: 0.12)	25.4 (se: 0.51)	25.5 (se: 0.43)	26.6 (se: 0.53)
Marital Status				
Single	21.0	30.0	26.9	18.2
Married	63.5	48.5	48.4	54.5
Living Together	11.7	12.1	15.1	16.4
Divorced	4.1	6.1	7.5	7.3
Widowed	0.2	3.0	2.2	3.6
Education Level				
No Education	11.0	9.1	11.6	14.5
Some Primary School	77.2	80.3	74.7	81.8
Some Secondary School	11.9	10.6	13.7	3.6
Religion				
Muslim	38.2	47.0	41.9	38.2
Catholic	43.7	43.9	48.4	45.5
Other/None	18.1	9.1	9.7	16.4
Intervention Group				
High Value	25.6	31.8	30.9	18.2
Low Value	27.5	33.3	33.0	41.8
Control	46.9	34.9	36.2	40.0
HIV/STI status at baseline				
HIV-pos at baseline	3.5	3.0	6.3	3.6
STI-pos at baseline^c^	16.1	7.6	12.6	16.4

a 59 of the 95 qualitative participants interviewed at 4 months were also interviewed at baseline

b 37 of the 55 qualitative participants interviewed at 8 months were also interviewed at baseline and 4 months, 9 were also interviewed only at 4 months

c Based on STI tests for Chlamydia, Gonorrhea, Syphilis and Trichomonas

The qualitative coding resulted in several categories of strategies to avoid unsafe sex, mentioned both at baseline and at the follow-up interviews. These included abstinence or periodic abstinence, having one partner who has tested and not using condoms, having one partner only and using condoms with this partner, convincing your spouse to use condoms outside of the marriage, convincing your spouse not to go outside the marriage, using condoms with your spouse, avoiding situations and circumstances that might lead to unsafe sexual behavior, filling time with other activities, reducing the number of sexual partners overall, having less risky partners, using the money to help convince a partner to stay safe, and separating or divorcing a current partner.

Within these categories of risk reduction strategies that were coded, three prominent themes emerged from the data as participants discussed how they adapted these strategies to avoid risk in the context of their daily lives, and in the context of the RESPECT study. First, we introduce the centrality of regular STI testing as a reliable method of overcoming the barrier of risk assessment and discuss the combination of targeted condom use, STI testing and treatment as a three-pronged approach to decreasing risk of infection. Second, we describe how certain situations provide increased leverage for sexual negotiation. Risk reduction strategies are often opportunistically implemented in these situations. Third, we explore the use of temporary abstinence as a frequently mentioned risk reduction strategy. What emerges from the data is that the barriers described in the previous section are addressed through innovative means of risk reduction. There are difficulties in assessing risk, but frequent and strategic STI testing can be used to ease those difficulties. Furthermore, women who otherwise lack agency in sexual decision-making as a result of marital and/or economic constraints can take advantage of new opportunities where they have leverage (enabled by both the cash awards and the new information about their sexual health from the STI testing) to minimize risk at certain points in time.

### Importance of STI Testing

The difficulty of assessing risk of a potential or current partner, in conjunction with the generally negative attitudes toward condom use creates a situation in which testing for HIV or STIs becomes an important tool in minimizing exposure to infection. For people who are in the early stages of a relationship, determining when a partner is safe, and when condoms are no longer needed is not straightforward. Testing has helped individuals in the RESPECT trial in making this determination. In fact, one strategy mentioned frequently during the interviews was a combination strategy involving condom use and testing for HIV and/or STIs. For those who are married or in long-term relationships for whom condoms are no longer a realistic option, regular testing provides an opportunity to assess risk and to bring temporary security (or insecurity, depending on the results) to relationships that are often plagued with uncertainty. This 29 year-old divorced woman (R for respondent) describes to the interviewer (I) how she and her partner negotiate risk by relying on the regular STI testing that the RESPECT study provided.

*I: Do you think you will continue using condom until in round four or you will use another strategy to make sure that you receive award?*

*R: We can use condom with him until there is time to examine his health…*

*I: Therefore, do you think that you will continue using condom or you will tell him that you are OK and hence there is no need to use condom?*

*R: I will trust him if he will be faithful and if he will protect his health. I can do sex without using condom when I am assured that we have tested and we are OK.*

-*Divorced woman, cash award group, 8-month visit*

It should be noted here that repeat testing in the context of the RESPECT study and as mentioned by the study participants during the in-depth interviews refers mostly to STI testing. HIV tests were performed only at the baseline visit and the 12-month visit of the study while STI tests were performed at all study visits (4 times in total). What emerged as particularly important to the study participants was the regular opportunity to learn about their health status. Every four months all study participants received information about their own health and their partner's health if their partner was enrolled in the study and willing to share their results. These results, as the data illustrate, translated into information about the risks participants face in their relationships. So while HIV testing is important, and in some ways the ultimate test of health status and risk exposure, the opportunity to repeatedly check health status using a proxy measure for sexual risk was of paramount importance.

The qualitative data also reveal that condom use is frequently viewed as a temporary strategy for risk reduction until some preferred strategy is made available. Condom use is often situational, sometimes based on objective evidence of the risk level of the partner, and sometimes based on a general feeling of trust. This man in the cash award group discussed how he and his partner use condoms between opportunities to test, especially when one of them has been away from the home for some time.

*I: When your partner went for testing she was negative. You still use condom even you are both negative?*

*R: We still use condom because of the opinion of the woman. When one goes away you have to use condom until when you test again and confirm that you are both OK.*

-*Unmarried man, cash award group, 4-month visit*

Some participants discussed how they find it difficult to trust potential partners when they say they have tested negative, and show concern if the most recent test was several months ago. Others mentioned that they were fine with just a verbally communicated result. . To show how study participants negotiate risk, we illustrate with several examples. The first comes from a 28 year old unmarried man in the low-value cash award group who used condoms with his partner until they could both get tested with good results.

*I told her to use condoms because before we married she was at her place and I don't trust her. Therefore, before I paid the dowry I wanted to use a condom until we shall go for medical check up. After the check up if our results are OK then we can continue doing sex without using condom. It was good because we came here for medical check up and since we are both OK we continued without using a condom.*

–*Unmarried man, cash award group, 4 month visit*

Testing to assess risk is not a strategy limited to partner selection, but is also used to assess the risk level of a current partner, as is illustrated above, or to prove to a partner that he or she is in fact risky. This interview excerpt is from a 29 year-old married woman enrolled in the RESPECT study.

*I: Did you talk with him when you came here for the first time and got your results?*

*R: Yes, I talked with him that my results were good and I also advised him to go for check up because my results were good. I advised him but he refused.*

*I: Now that you have received your results today will you talk with him about the kind of results you have got?*

*R: I will not tell him because if I am infected it means that he is the one who transmitted this to me. This is because maybe he is the one infected and had he come for tests this disease would have been treated already. Therefore, when I am going he must go for test and if he doesn't want to come here I will tell him to go to the hospital*

*I: Do you think he will accept when you tell him to go for testing?*

*R: This time he will accept to go to test*

-Married woman, cash award group, 4 month visit

This passage highlights the centrality of testing as a risk reduction strategy within a marriage, where condom use is not an option. This woman discusses the importance of having the actual test results in hand so that she can use them as leverage to get her husband to test, and as a result of his test results, she can convince him to change his behavior. The power of persuasion in this case lies in the test results and the ability to show a partner that he or she is risky with authority.

Our data reveal that regular testing alone and in combination with condom use is a risk reduction strategy used to facilitate the problematic issue of understanding the risk level of a current or potential partner. The information that test results provide brings some certainty to relationships that, as regards risk of infection, are often filled with ambiguity. The new information and knowledge that comes from test results can also create opportunities for negotiating safer sex where previously little agency in decision-making existed—testing is important both for risk assessment as a bargaining chip in sexual negotiations.

### Situational Leverage and Opportunistic Implementation

A second insight that came out of the qualitative data and the discussions about strategies to avoid unsafe sex related not to a specific strategy, but rather to how and when strategies were implemented. Risk avoidance was often practiced inconsistently and episodically. This was especially true for women who faced the barriers discussed in the Introductory section; women who much of the time lacked sexual decision-making power in their relationships. During their interviews, these women talked about how they took measures to reduce their risk of infection when they felt they could–if an opportunity arose that temporarily gave them increased agency, they took that opportunity to protect themselves. These opportunities included circumstances or situations that provided women with increased knowledge, and as a result, added leverage with which to negotiate with their partners. Specifically, added leverage arose within the RESPECT study from the cash incentive and STI testing status knowledge. More broadly outside the context of RESPECT, other situations could provide opportunities for women to refuse sex or enforce condom use—such as after having a baby, or when a husband or partner felt guilty about being with another woman. One 19 year-old married woman explained how she is sometimes able to convince her husband to use condoms.

*I: Did you try to avoid unsafe sex?*

*R: Yes*

*I: What did you do?*

*R: I asked my husband to use condom the days when he was not in good mood*

*I: Did you use it throughout or in dangerous days only*

*R: During dangerous days only*

*I: What are these dangerous days?*

*R: When my husband comes back at 2 am in the night and he needs to stay with me I ask him to use*

*I: Is this because you don't trust him?*

*R: Yes*

*I: So, you think this time you can get STI?*

*R: Yes*

*I: But when he comes back early you continue as usual?*

*R: Yes*

*I: Can’t you get STI when he comes early? What do you think?*

*R: I can*

–Married woman, control group, 4 month visit

One possn is taking action when she feels she has leverage to take action. She understands that she can still be infected if she uses a condom today but not tomorrow, but she also understands that she needs to be strategic about when she can implement her prevention efforts. Another possible interpretation is that she is angry about her husband staying out late with other women and when this situation presents itself, it gives her an opportunity to confront him and perhaps to discourage his infidelities.

Some women used the RESPECT award cash to help extract themselves from risky sexual behaviors and relationships that had been driven partly by economic constraints. This woman who lives with her partner talked specifically about how the cash award associated with the study has allowed her to leave other men because the money from the study will help her in her life.

*R: I have come back for round two of this study because when I come back I learn more about my health…another thing is the award, when I came I was given award and I know what to do with it so as to take care of my children. The prostitution behavior I had before, I can stop it completely*

*I: You were a prostitute…can you explain to me what kind of a prostitute were you?*

R: Changing men from time to time. All this was because of problems

*I: Did you have the behavior of having many men before you were enrolled to this study?*

*R: Yes, I had this behavior before I was enrolled to this study*

I: Over the period of four months ago have you had multiple sex partners?

*R: I didn't have this behavior; I left them completely…since I knew my health status and got the award I decided to continue with my life and stop this behavior; I have known that my health is OK…Therefore, when I get here there will be money for inconvenience to use with my children. So, what is the importance of continuing with these men?*

–*Married woman, cash award group, 4 month visit*

Others used their enrollment in the study as a leverage point to achieve goals that they had previously (such as getting their husbands to be more faithful), and were able to successfully avoid sex or enforce condom use as a result. Joint strategizing between couples enrolled in the study was also mentioned by both men and women in the cash award groups. If a woman's partner or spouse is enrolled in the study, the couple might discuss staying safe together so that both of them can receive the award, and the woman might have more leverage, backed by the money that they will both receive, to convince her husband to leave outside partners or use condoms with outside partners. This example from a woman in the cash award group illustrates how enrollment in the study and the promise of the reward provided an opportunity for her to discuss the issue of safer sex with her husband.

*R: I told him we are supposed to go for check up, at the first round we shall check blood and STI and after four months we shall come again for testing and if we test negative we will get reward.*

*I: Therefore, you discussed together about what you should do in order to get reward?*

*R: yes we discussed together…I told him after four months if I test negative for STI we shall get reward. Therefore, I told him to take care and he was ready*

*I: Did he listen to you?*

*R: He listened to me so attentively*

*I: Did he accepted to take care?*

*R: Yes*

-*Married woman, cash award group, 4 month visit*

Outside of the RESPECT intervention mechanisms, other points of leverage for women to gain some agency in sexual decision-making include recently having had a child, or being infected with an STI. Because it is common not to resume sex for some time after a baby is born, women who have just given birth are in a position of having the ability to refuse sex without consequences, and can then attempt to extend this abstinence—though only temporarily. Having this temporary power to refuse sex with husbands may be especially important for married women who know that their husbands have other partners, but are not sure if he uses condoms with his external partners. Adding the RESPECT STI testing to this situation could further help women convince husbands that there could be real consequences to his own risky behavior during her postpartum abstention.

### Temporary Abstinence

Temporary abstinence is a risk reduction strategy that, depending on the circumstances, is likely to be limited in its effectiveness in preventing HIV infection. However, what emerges from the data is that given the range of strategies available, individuals in this setting are at least as likely to rely on temporary abstinence as they are on condom use. Temporary abstinence could include divorce (in its most extreme form), temporary physical separation from a partner, enforcing no sex for several months after the birth of a child, enforcing no sex after a positive STI test, or enforcing no sex because of the recognition that a husband has been out with another woman.

If discussions of condom use, either within the marriage or with external partners, were ineffective, one option is to physically separate themselves from their husbands temporarily to lower risk. In the case of this 27 year-old married woman, simply the threat of separating their beds convinced her husband of the importance of avoiding having external partners.

*I didn't know what to do because at times he was coming back home at 11 pm in the night and when I asked him where he was he said that he was working. I warn him if he was moving around but he told me that he was still working. I told him that if it doesn't work out we shall separate our beds so that everyone sleeps alone. He listened to me and that is why I tested negative today*

–*Married woman, cash award group, baseline visit*

The negative test results were enough to convince this woman that her husband had listened to her and was not “moving around” (engaging in sexual relationships with other women). This example again illustrates the importance of the test results in establishing trust with a partner—either for partner selection, or building trust with a current partner. For others though, a test result is not enough to convince a partner to change his or her behavior, or even to get tested. In this case, physical separation from a partner is sometimes the only option.

This passage from one of the diaries in May of 2009 documents a conversation between two women, one of whom is convinced she has an STI and wants her husband to get tested so they can both take treatment.

*When I go to the working place on foot, I saw two girls about 30 years they were in their own talks. After a time first girl said to her fellow that “aiseeh! Everyday I talked to your relative about my health, but he didn't want to understand me, please go and advise him, I am ready start to use a dose but not him. And when I advise him to go and to check it [his health], he didn't. When I want to use condom during sex he refuse to do it. So it's your time to educate him. Nevertheless I will go back home even without permission from him.” The second girl agreed and adds that, “even me I amazed from him, now is the world of truth in marriage, ok I will try to advice him and if he refused! Even me I will support you to go back home– that is not life.” *(Diary, after baseline visit)

This example uncovers two alternative strategies–physical separation from an uncooperative husband, but also using social networks and connections to help with convincing a partner to understand the consequences of his actions and perhaps change as a result.

The next step after temporary separation is permanent separation or divorce–another form of temporary abstinence, in many cases a more realistic strategy for risk reduction, for both the wife and the husband, than convincing a partner to use condoms within the marriage. Work done in Malawi has shown that divorce has increasingly become more common overall, and marital dissolution has increasingly been implemented as a strategy to protect oneself from an unfaithful spouse and from HIV [11], [38], [39]. In fact, as HIV/AIDS became more prevalent and was perceived as more of a threat, divorce as a response to infidelity was also steadily increasing in Malawi [39]. Over time, the proportion of women who agreed that divorcing a husband who was unfaithful or was suspected of having HIV was justified also increased significantly based on these data from Malawi [11].

Both women and men enrolled in the RESPECT study discussed having used this strategy in the past to separate themselves from a partner they perceived as risky. Female participants in the cash award groups also sometimes discussed divorce as an extreme strategy they might need to implement in order to stay safe within the context of the RESPECT trial, and to ensure that they would be eligible to receive the award—though this seems unlikely given that participants were only enrolled in RESPECT for one year. Other women are clear that they do not see divorce as an option at all, and are resigned to the reality that facing risk from their husband is part of the marriage experience. This 29 year-old woman left her first husband (in the past, prior to the RESPECT trial) because she was worried that he would bring infection into their marriage.

*In the past I would fear but since last year I was not worried to say that I have infections because I divorced my husband. I stayed for 5 years and I got another husband. I refused to accept him for quite long time but he told me that he was OK. We decided to go for testing and we found that both were OK. I stayed with him and I gave birth to one child who is 2 years and 2 months old. Therefore, since I gave birth to this child I stayed without doing sex and I didn't do sex even with the one I have this child. I decided to leave him because his service was not good. Therefore, I saw there was no meaning to stay with such a person.*

–*Divorced woman, cash award group, baseline visit*

This next woman also appears serious in considering divorce as an option as she exploits the new opportunity arising from RESPECT′s STI testing program to convince her husband to either leave his external partners, use condoms with his external partners, go for testing, or use condoms within the marriage. This married woman discusses her frustration in trying to convince her husband to either use condoms with her in the marriage or to go for testing.

*R: The new strategy is to use condom and if he doesn't want…But the first strategy is to tell him to go test and I should make sure that I go with him if he accepts to go for testing. If it will be OK we will do safe sex.*

*I: Do you think he will agree with this?*

*R: He will accept…I don't know what I will do if he refuses; it is better to divorce than to get diseases…I will continue advising him to come for testing in round three. If he refuses to come I am ready to divorce him rather than getting STI*

–Married woman, cash award group, 4 month visit

It is notable that safe sex in this excerpt, and often in the interview transcripts, refers not to sex using a condom, but sex with someone who has proven through testing or through verbal acknowledgment that they are safe.

Another woman discussed her difficulty in trying to convince her husband to use condoms even though she knew that he had other women. Part of the difficulty, as she mentions, had to do with the expectations around having children and how using a condom when more children were expected was not an option.

*I: How do you feel now that you that you do not have STIs or HIV?*

*R: I feel so much by knowing this than just staying without knowing my health status.*

*I: Will this make you change your behavior?*

*R: This has made me change my behavior quite much to the extent that it made me leave my husband because he was just talking about these things but he was not ready to change his behavior. I decided to leave him because I advised him so much but he didn't want to change. Since we are a husband and wife I cannot tell him to use condom because we are still procreating. We have to do sex without using condom. Since you do not want to change then you can cause death to me (diseases). This is what made me leave him.*

-*Married woman, cash award group, 8-month visit*

For other women, divorce or separation is not part of the menu of available strategies. They may be financially constrained from leaving their husbands, or because of the emotional attachment leaving may be too difficult.

*I: Do you think this time your husband will accept to use condom?*

*R: I don't know if he will accept, if he refuses that is it*

*I: What will you do if he refuses?*

*R: What will I do; I am in the marriage*

*I: Do you have freedom to tell your husband that if he does not put on condom you will not do sex with him?*

*R: Yes, I will refuse…because I will refuse today…tomorrow I will refuse…the day after tomorrow I will refuse… at the end I have to agree, I have to do it without condom…this man is just like that, you can tell him this today and he can accept, but he can refuse tomorrow.*

–*Married woman, cash award group, 4-month visit*

Younger, single men often discussed exercising sexual control through avoidance—either avoidance of sex or avoidance of situations that might lead to risky sex, for example those with alcohol. Another path of avoidance mentioned frequently was keeping oneself occupied with other activities, such as exercise, studying, and or working on their farm, so that little time remained to focus on meeting women and sex. Such avoidance was preferable to consistently using condoms with partners–these men found it more realistic to avoid sex than to trust themselves that they would use a condom every time. This excerpt from a single man enrolled in the RESPECT study illustrates these strategies.

*I: What strategies did you use in order to get the reward?*

*R: The first thing is to come back home early, not staying with people with bad behavior…some people want to stay with ladies all the time, when they sit somewhere they are talking about sex only. Now I am avoiding such people…because when you join their company anything can happen.*

*I: Will it be easy for you to avoid these temptations?*

*R: Yes, it is easy…when I think that my friends are about to come I can go somewhere, I can decide to go to shamba [the farm] to look at my maize. Then I decide to leave, when they come they will be told that I am not around*

–*Unmarried man, cash award group, 4 month visit*

What emerges from these data are that in this context, while episodic use of condoms and abstinence are likely similar in their limited effectiveness in HIV prevention, individuals in this setting are at least as likely to rely on temporary abstinence than they are on temporary condom use. Temporary abstinence is not a reliable means of preventing infection. However, refusing or avoiding sex at certain opportune moments or under specific circumstances is an intermediate strategy that over time might lead to more permanent strategies for risk reduction. If, for example, enforcing temporary abstinence by sleeping in separate beds or refusing sex if a partner comes home late at night is perceived as punishment, this intermediate strategy could eventually lead to a partner changing his behavior. Sexual behavior change is slow at best, and implementation of intermediate strategies such as temporary abstinence is both a means of gaining transient control, and perhaps a method of pushing for change through increased control.

## Discussion

By exploring how study participants responded to an economic incentive to remain STI negative, this analysis adds to our current understanding of approaches to risk reduction by highlighting the often opportunistic and episodic implementation of strategies by women who face behavior change constraints [40]. Women who may in typical circumstances lack sexual decision-making ability can and often do take advantage of situations that present added leverage with which to negotiate.

The analysis is particularly helpful for understanding mechanisms through which behavior change occurred in the RESPECT trial. Both the cash awards and the receipt of STI test results through RESPECT provided opportunities for attempting behavior change. Simply being enrolled in a study that repeatedly provided the ability to check one's health status, and the promise of the cash incentive for testing negative for those in the cash award groups, provided participants with added negotiating power in their sexual relationships. In fact, the repeated testing became part of the strategy in some cases as the combination of targeted condom use, testing and treatment was sometimes implemented as a three-pronged approach to decreasing risk of infection. Having a back-up for condom use is important in a society where condom use carries with it such strong meaning about the type of relationship [12]. In addition, having the ability to use testing as a milestone after which condoms are no longer necessary provides some leverage for convincing a partner to use condoms.

Temporary abstinence, in its many forms, also emerged as a more prevalent strategy than expected. The apparent popularity of this approach may be a result of the negative associations with and low levels of use of condoms within long-term serious partnerships. The data presented here suggest that abstinence is a favored strategy over condom use in certain situations–among young single people, among women who have just had children, and among women who can argue that they have been put at risk by their husbands. Married women seem to be more likely to be able to abstain from sex with their husbands in order to at least temporarily reduce their risk of infection than they are to insist on condom use. This is of course an imperfect strategy for protecting their own sexual health, thus it will be important for future educational efforts to ensure that women understand the limited situations in which this can protect them from infections.

The present analysis was not designed to definitively establish the relative importance of cash awards versus STI testing in providing opportunities and tools for behavioral change. The quantitative evaluation results previously published indicate that RESPECT′s high value cash award arm did experience 27% fewer STIs than the control arm; since testing was available to both arms, this difference is likely attributable to the cash awards [9]. That prior analysis was not able to quantify the effects of the STI testing regimen per se (the study was not designed to directly do so), thus the present analysis is particularly important for uncovering a strong latent demand for STI testing. It is also reasonable to speculate that the testing demand was enhanced by the cash awards, and that conversely the effects of the cash awards may also have been enhanced by the behavioral change opportunities afforded through the testing. Thus cash awards and STI testing may well be synergistic components of the RESPECT package – just as these may have enhanced (and in terms been enhanced by) the effectiveness of traditional behavioral change communication campaigns.

Lessons for adopting STI testing interventions beyond a RESPECT-style intervention are more difficult to ascertain. Regular, comprehensive STI testing is a costly intervention; alternatively, using a less comprehensive testing regimen could undermine women's ability to use the testing for the purposes of risk assessment (and could even raise risks due to inaccurate infection information). STI testing has often been considered an intervention targeted at identifying positive individuals so as to provide them treatment (or more controversially, to reduce HIV transmission probabilities [41]), which may be difficult to justify on direct cost-benefit grounds. But the present study does suggest that beyond this direct epidemiological value, STI testing may have important behavioral implications as well, through its role in providing opportunities and leverage for behavioral change. This behavioral pathway merits careful future research.

A deeper understanding of how cash awards and STI testing was perceived and acted on would help in structuring related new intervention models. The opportunities provided by the RESPECT study may have altered perceived behavioral control and self-efficacy among women enrolled in the trial, thus temporarily facilitating behavior change [42], [43]. Our results demonstrate the fact that individuals in this setting are willing to adopt temporary risk reduction strategies even if they fall short of consistent behavior change. Future work would also be useful to further explore how these opportunities and strategies may have either been stymied among women facing gender-based violence, or alternatively helped to overcome such barriers. Related analysis has documented a decline in gender-based violence during the course of the 1-year RESPECT study [44]; future research examining varying risk change strategies by baseline violence level would be illuminating.

This study brings with it some limitations. As with any study that includes self-reported sexual behavior as a data source, there is the possibility that unsafe sexual behaviors were under-reported. Plummer et al report on the validity of the collection of sexual behavior data using five different methods from a study done in Northern Tanzania [16]. While this study was conducted amongst adolescents, and results among adults may be slightly more congruous across the five methods, they find striking inconsistencies in reports of sexual behavior from self-administered surveys, face-to-face surveys, in-depth interviews, participant observation and biological markers. Social desirability bias is common in these types of studies, and in this case may have been exacerbated by the extended counseling on safer sex practices that participants were receiving throughout the study.

An additional limitation relates to the translation and transcription of audio-taped interviews. There is the potential for the content of the interview to lose meaning and nuance during the transcription and the translation process. We addressed this limitation to some degree by having one of our study interviewers (who is bi-lingual) review the transcripts and translations within two weeks of the actual interview, and revise the transcripts as necessary. This method still suffers from a secondary but related limitation; that is the revised translated transcripts are an interpretive process, wholly dependent on the perception of the interviewer. Limitations related to the use of the village diaries should also be noted. The pay for the diarists may have motivated them to seek out situations in which HIV and/or the study is being discussed [36]. Bias may also result from the diarist's individual perceptions of what he/she is hearing, and bias resulting from potentially inexact recall of the situation may also arise. However, it is also important to emphasize the purpose of this data is to provide a window into what is going on in the community from the community perspective, fully understanding that we are gaining this information through the lens of one of the members of the community.

Loss of the 14 of the qualitative transcripts during the first round is an additional limitation of the study. The recordings were inadvertently deleted while the team was out in the field and could not be recovered. While this does not pose a problem in relation to bias since there was no systematic loss of data, it is a limitation in that we lost data that would have contributed to our findings and results, but the limitation is minor given the large number of qualitative interviews analyzed. The purposive sampling to select more candid respondents in the subsequent follow-up visits is an additional limitation in that this type of selection may have introduced a bias. By selecting those respondents who were more open to discuss their strategies for avoiding unsafe sex, we may have also selected respondents utilizing particular types of strategies over and above other strategies. This may have systematically influenced our findings, although we have no evidence of this happening.

Our data suggest that when opportunities to implement risk reduction strategies present themselves, they are regularly taken. The qualitative data point to the importance of not only the cash incentive, but also the access to regular, reliable testing and knowledge of health status in opening opportunities to discuss risk reduction strategies with partners and as leverage in negotiating and implementing risk reduction strategies. With the understanding that behavioral change strategies are a necessary but not sufficient means of preventing HIV, the RESPECT approach may have acted as a combination HIV prevention intervention. Our results suggest that RESPECT′s structural cash intervention and the testing component worked synergistically to assist men and women with opportunities to better act on behavioral change intentions, thus potentially increasing the effectiveness of traditional behavior change interventions as well.
